# In-Depth Characterization of Two Bioactive Coatings Obtained Using MAPLE on TiTaZrAg

**DOI:** 10.3390/ma17122989

**Published:** 2024-06-18

**Authors:** Mariana Prodana, Andrei Bogdan Stoian, Daniela Ionita, Simona Brajnicov, Iulian Boerasu, Marius Enachescu, Cristian Burnei

**Affiliations:** 1 Department of General Chemistry, Faculty of Chemical Engineering and Biotechnologies, National University of Science and Technology Politehnica Bucharest, 313 Splaiul Independentei, 060042 Bucharest, Romania; mariana.prodana@upb.ro (M.P.); andreibstoian@yahoo.com (A.B.S.); 2 Lasers Department, National Institute for Lasers, Plasma and Radiation Physics, 409 Atomistilor Street, 077125 Magurele, Romania; brajnicov.simona@inflpr.ro; 3 Center for Surface Science and Nanotechnology, National University of Science and Technology Politehnica Bucharest, 313 Splaiul Independentei, 060042 Bucharest, Romania; iulian.boerasu@cssnt-upb.ro (I.B.); marius.enachescu@cssnt-upb.ro (M.E.); 4 Clinical Department of Orthopedics and Traumatology II, Clinical Emergency Hospital, Calea Floreasca 8, 014461 Bucharest, Romania; cristian.burnei@umfcd.ro

**Keywords:** MAPLE, chitosan, bioglass, bioactive, titanium alloy

## Abstract

TiZrTaAg alloy is a remarkable material with exceptional properties, making it a unique choice among various industrial applications. In the present study, two types of bioactive coatings using MAPLE were obtained on a TiZrTaAg substrate. The base coating consisted in a mixture of chitosan and bioglass in which zinc oxide and graphene oxide were added. The samples were characterized in-depth through a varied choice of methods to provide a more complete picture of the two types of bioactive coating. The analysis included Fourier transform infrared spectroscopy (FTIR), scanning electron microscopy (SEM), ellipsometry, and micro-Raman. The Vickers hardness test was used to determine the hardness of the films and the penetration depth. Film adhesion forces were determined using atomic force microscopy (AFM). The corrosion rate was highlighted by polarization curves and by using electrochemical impedance spectroscopy (EIS). The performed tests revealed that the composite coatings improve the properties of the TiZrTaAg alloy, making them feasible for future use as scaffold materials or in implantology.

## 1. Introduction

TiZrTaAg and other Ti-based alloys are remarkable materials with exceptional properties, making them noteworthy candidates for use in medical applications [1] and various industrial fields like the general automotive industry or energy production [2,3]. The metals used for the fabrication of this alloy possess high strength and low density, making the TiZrTaAg alloy an ideal choice for structural applications [4]. This superior combination of elements results in an alloy that holds immense potential in engineering, medical, and aerospace fields [5]. One of the remarkable properties of the TiZrTaAg alloy, like other titanium alloys [6,7,8,9], is its impressive corrosion resistance. This alloy can withstand harsh environments with ease, making it suitable for use in marine, chemical, and thermal power plants. Its resistance is enhanced by the addition of Zr and Ta, as they form a stable oxide layer on the surface, preventing any further corrosion. In the medical field, the TiZrTaAg alloy has gained significant attention for its biocompatibility and biostability [10]. These properties are crucial in the development of medical implants and prostheses. The biocompatibility of titanium-based alloys means that they do not cause any harmful effects when in contact with living tissues, making them suitable for use in implants such as dental implants, joint replacements, and bone fixation plates [11,12,13]. Their biostability ensures that the implants remain intact and functional for a long time without causing any adverse reactions in the body.

The Matrix-Assisted Pulsed-Laser Evaporation (MAPLE) deposition technique is a cutting-edge technology that has revolutionized the way thin films and coatings are deposited on different substrates [14]. MAPLE uses laser energy to transfer thin films of different materials while maintaining the original characteristics of the material [15]. MAPLE deposition technique offers a high degree of control over the deposited particles’ size, shape, and orientation, which is critical for many advanced applications [16,17,18]. By adjusting the laser energy and duration, the thickness and composition of the deposited film can also be controlled precisely. This level of control and precision is not achievable with other conventional thin film deposition techniques, making MAPLE deposition a preferred method for many researchers and scientists. Thin films are gaining a lot of attention in the field of material science due to their unique properties and diverse applications. Chitosan (Chi), a naturally occurring polysaccharide derived from the shells of crustaceans, is widely known for its biocompatibility and biodegradability. It has been extensively studied for its potential use in pharmaceutical, biomedical, and environmental applications [19]. Chitosan has a unique structure that allows it to form a thin film when coated on a substrate. Its film-forming properties, along with its biocompatibility, make it an ideal component for thin films. Bioglass (BG) has gained popularity due to its ability to integrate with bone tissue, making it an excellent candidate for bone regeneration applications [20,21]. When combined with chitosan, the resultant thin film not only has the mechanical strength provided by chitosan, but also the bioactivity of bioglass, making it a perfect solution for bone tissue engineering. Zinc oxide (ZnO) is a multifunctional material that is widely used in various fields [22]. Its unique properties, such as high thermal stability, transparency, and biocompatibility, make it a suitable component for thin films. When incorporated into chitosan–bioglass thin films, it adds to the mechanical robustness and provides antibacterial and anti-inflammatory properties. Graphene oxide (GO), on the other hand, is a two-dimensional material with exceptional strength and electrical conductivity [23]. It has been extensively studied for its potential applications in electronics, energy storage, and biomedical fields. When combined with chitosan–bioglass thin films, it enhances the bioactivity of the film. The combination of chitosan, bioglass, zinc oxide, and/or graphene oxide in thin films has opened a whole new area of research and development. These films have a wide range of potential applications, including wound healing, drug delivery, tissue engineering, biosensing, and antibacterial coatings. Furthermore, these thin films offer some distinct advantages over conventional materials. They have excellent mechanical stability, and can be tailored for specific applications, having enhanced biocompatibility and bioactivity. The ability to control the thickness, composition, and surface properties of the thin film makes it a versatile material for various applications.

Studies performed on other alloys in NaCl revealed that the corrosion processes start at the sites of micro-defects present on the surface of the metal [24,25], and that amorphous coatings rich in nitrogen can improve their corrosion resistance [26].

In the present study, two types of bioactive coatings using MAPLE were obtained on a TiZrTaAg substrate. The base coating consisted in a mixture of chitosan and bioglass in which zinc oxide or graphene oxide were added. The samples were in-depth characterized through a varied choice of methods to provide a more complete picture of the two types of bioactive coating. Starting from a previous study [1], new coatings based on chitosan and bioglass were developed in which ZnO or GO were incorporated. The electrochemical, physical, and mechanical properties were determined, and the results were used to observe the effect of adding ZnO or GO in the chitosan and bioglass matrix.

The aim of our study is to develop new bioactive organic/inorganic coatings, using MAPLE deposition, that can be used as joint or bone replacement. The organic part (Chi) will provide a matrix for the inorganic fillers (BG, ZnO, and GO) and will enhance the adhesion between the titanium alloy and the coating. The inorganic part such as BG, ZnO, and GO presents antibacterial properties, and can also improve the mechanical properties of the coatings.

## 2. Materials and Methods

The suspensions to be deposited using MAPLE on TiZrTaAg substrates were prepared using chitosan (190–310 kDa, 78–85% deacetylation, Sigma-Aldrich, Saint Louis, MO, USA), pure water (Purelab Option R7BP, ELGA, High Wycombe, UK), acetic acid (Sigma-Aldrich), bioglass (BG-45S5), ZnO nanoparticles, or graphene oxide (Sigma-Aldrich). The bases of the coatings were formed by a solution of chitosan with a concentration of 0.5 g/L dissolved in 1% aqueous acetic acid solution, and after its dissolution, nanoparticles of BG, ZnO, or GO were added in various mass ratios. All prepared suspensions were magnetically stirred for 5 min followed by ultrasonication for 30 min and magnetic stirring again for 15 min to achieve an adequate dispersion of the suspension components. 

The alloy samples (73Ti-20Zr-5Ta-2Ag) were polished with SiC paper (Buehler, Lake Bluff, IL, USA) ranging from P800 to P3600 grit. Subsequently, they were cleaned in an ultrasonic bath with acetone, ethanol, and distilled water for 10 min each before being dried in the atmosphere. The procedure for obtaining chitosan-based coatings using the MAPLE technique is briefly described. To fabricate the solid targets, the chitosan + BG + ZnO and chitosan + BG + GO mixture were suspended in water and flash frozen using liquid nitrogen, resulting in solid targets. Next, the frozen solid target was placed inside the vacuum chamber with a working pressure of 5 × 10^−5^ mbar. It was then exposed to a pulsed Nd:YAG laser beam (Surelite II, Continuum, Bordeaux, France) with parameters of λ = 266 nm, ν = 10 Hz, and τ = 5–7 ns. An optical system directed and concentrated the laser beam within the processing chamber, hitting the solid target’s surface at approximately a 45° angle of incidence. The laser was calibrated to a fluence of 2 J/cm^2^. 

The target–substrate distance was 30 mm, fixed throughout the deposition.

The samples presented in this work have the composition shown in Table 1.

Infrared spectroscopy (FT-IR) was carried out with a Perkin-Elmer Spectrum 100 instrument (Perkin-Elmer, Shelton, CT, USA), operating in absorbance mode, across the range of 600–4000 cm^−1^. The morphological aspect of the specimens was examined with scanning electron microscopy (SEM) using a Hitachi SU 8230 (HITACHI High-Technologies Corporation, Tokyo, Japan) at 10 kV, with a chamber pressure of 1 × 10^−4^ Pa, and a working distance of 9 mm. The microscope included an energy-dispersive X-ray (EDS) analyzer (Oxford Instruments, Abingdon, UK). Ellipsometry measurements were performed using a M-2000 Spectroscopic Ellipsometer (J.A. Woollam, Lincoln, NE, USA) system testing 3 different areas for each individual sample. The analyses were carried out at 3 different angles of incidence (60°, 65° and 70°), varying the wavelength of the beam in the range of 300 nm–1000 nm. The samples were analyzed structurally using micro-Raman spectroscopy technique at room temperature using a LabRam HR800 (HORIBA France SAS, Longjumeau, France) system. Each Raman spectrum was produced by subjecting samples to a 632 nm laser beam for 100 s with a power of 2 mW, then capturing the emitted signal in a CCD detector with a 600-line grating. Micro-Raman spectroscopy is also very useful in identifying the structure of the deposition. Vickers hardness was determined using a Wilson Tukon 1102 hardness system (Buehler, Lake Bluff, IL, USA) under 0.5–5 kgf load, over 10 s. To determine the Vickers hardness number, an average of the five indentations was used. The force–distance (F-Z) curves and the roughness of the samples were obtained using an A.P.E. Research A100-SGS atomic force microscope (A.P.E. Research, Trieste, Italy). This work shows the normalized average curves obtained from 5 measurements for each sample. The evaluation of corrosion rate and stability of the samples was carried out using electrochemical tests with a PGSTAT100N potentiostat (Metrohm Autolab, Barendrecht, The Netherlands) in 0.9% NaCl solution. Three-electrode cells were used, with the samples as working electrodes, Ag/AgCl as a reference electrode, and a Pt sheet as a counter electrode. Impedance spectroscopy was conducted at open-circuit potential (OCP) in the frequency range of 10^4^ to 10^−1^ Hz with an amplitude of 10 mV, while corrosion monitoring (Tafel plots) was carried out at ±200 mV compared to OCP.

## 3. Results and Discussion

### 3.1. FTIR Measurements

**Figure 1:**
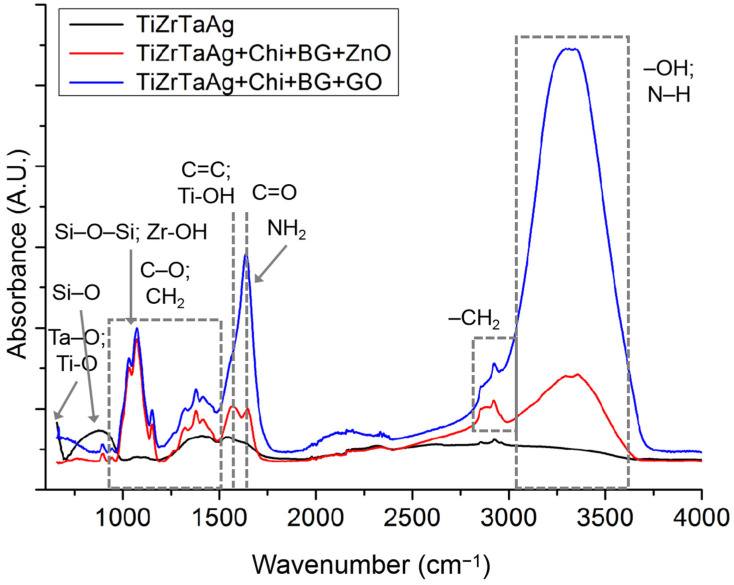
FTIR spectra for analyzed samples.

The FT-IR spectra obtained for every sample are displayed in Figure 1. The peaks in each sample are present at frequencies ranging from 3584 to 3700 cm^−1^ and 1310 to 1390 cm^−1^, which correspond to the stretch and bend vibrations of the -OH group from water. Si-O bonds in BG were observed at 800 cm^−1^ and 1000 cm^−1^, corresponding to Si-O vibration and Si-O-Si vibration, respectively. Chitosan exhibited a distinctive signal of amide I at 1637 cm^−1^ originating from the undecetylated portions of chitosan [27], amide II at 1548 cm^−1^, and amide III at 1323 cm^−1^. The frequencies observed at 1412 cm^−1^ and 1374 cm^−1^ correspond to the bending movements of the C–H_2_ and C–H_3_ chemical bonds. The bands from 1154 cm^−1^ to 1027 cm^−1^ are linked to the C–O–C of the glycosidic bonds. The bands detected at 1155 cm^−1^ and 800 cm^−1^ are identified as the C–O stretch vibrations of chitosan [28]. 

For Sample S1, the following peaks were attributed: Ti-OH stretching around 1600 cm^−1^, Ta-O stretching at 690 cm^−1^ overlapping the Ti-O stretching, and Zr-OH bending at 1120 cm^−1^. The presence of Zn in the coating of sample S2 was confirmed by the existence of a peak at 625 cm^−1^ and the increase in intensity of the peak at 3500 cm^−1^, due to the probable interactions between the OH, NH_2_, and ZnO. For sample S3 it is confirmed that the –OH and –NH groups are present, showing a stretching vibration of its band from 3400 to 3270 cm^−1^ and a bending vibration peak characteristic of –NH_2_ or –NH_3_^+^ at 1530 cm^−1^. A number of GO’s well-known characteristic peaks are visible, such as C=O at 1720 cm^−1^, the conjugated structure C=C at 1620 cm^−1^, the carboxyl group C–O at 1400 cm^−1^, and the epoxy structures C–O at 1220 cm^−1^) and C–O at 1050 cm^−1^.

### 3.2. SEM EDX

**Figure 2:**
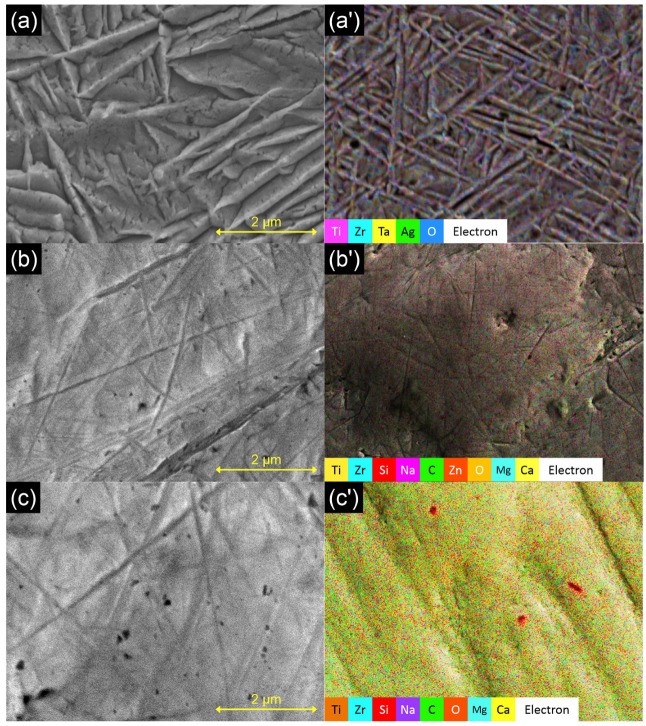
SEM and EDX mapping for samples: (**a**,**a’**) S1; (**b**,**b’**) S2; and (**c**,**c’**) S3.

On the surface of sample S1 (Figure 2a,a’), oxides with typical acicular structures found on Ti-Zr alloys can be seen. Rare silver nanoparticles formed of small, clustered spheres can be seen scattered on the surface being visible as bright dots on the SEM images and, more clearly, on the EDX mapping images at high magnification. The sample is homogeneous, with no agglomerations found on the surface of the sample.

Images obtained for samples S2 (Figure 2b,b’) and S3 (Figure 2c,c’) show that the films obtained using MAPLE are uniform, covering, for the most part, the imperfections of the native substrate surface. The presence of the narrow canals that can be seen on the surface of the samples hints that the deposited film has varying thicknesses. This morphology is typical of polymeric depositions using MAPLE, and is called in the specialized literature “see-island-like morphology”. The islands have diameters between 20–50 μm on both coated samples. On sample S2, pores of around 1–3 μm were found scattered on the surface. On sample S3, fewer pores were found, and the diameters were also smaller, reaching only around 1 μm.

Elemental mapping images and composition (Table 2) revealed elements both from the alloy substrate and from the film components present on the investigated samples.

### 3.3. Ellipsometry

The spectroscopic ellipsometry data (Ψ and Δ) have been experimentally recorded on the surfaces of samples S2 and S3 at an angle of incidence of the light beam of 60, 65, and 70, in the spectral range from 370 nm to 1000 nm. 

For simulating the ellipsometry data, two different models were considered. The first one was applied in the case of the S2 sample by considering two different materials, i.e., the alloy material (as substrate), and the Chi + BG + ZnO blend material (top layer). The second used model was applied to the S3 sample, and considered the alloy material to be a substrate and the Chi + BG + GO blend material to be the top layer.

For modeling the recorded data, a mixture of voids with chitosan-based blends in equal proportion of (30:70) were also considered in both developed simulations. The Bruggeman and Maxwell–Garnett algorithms were applied for estimating the optical constants and the thickness of the deposited layers is determined. According to the calculus results presented in Table 3, the thickness (h) of the coating including GO is slightly higher than those with no GO.

### 3.4. Micro-Raman

**Figure 3:**
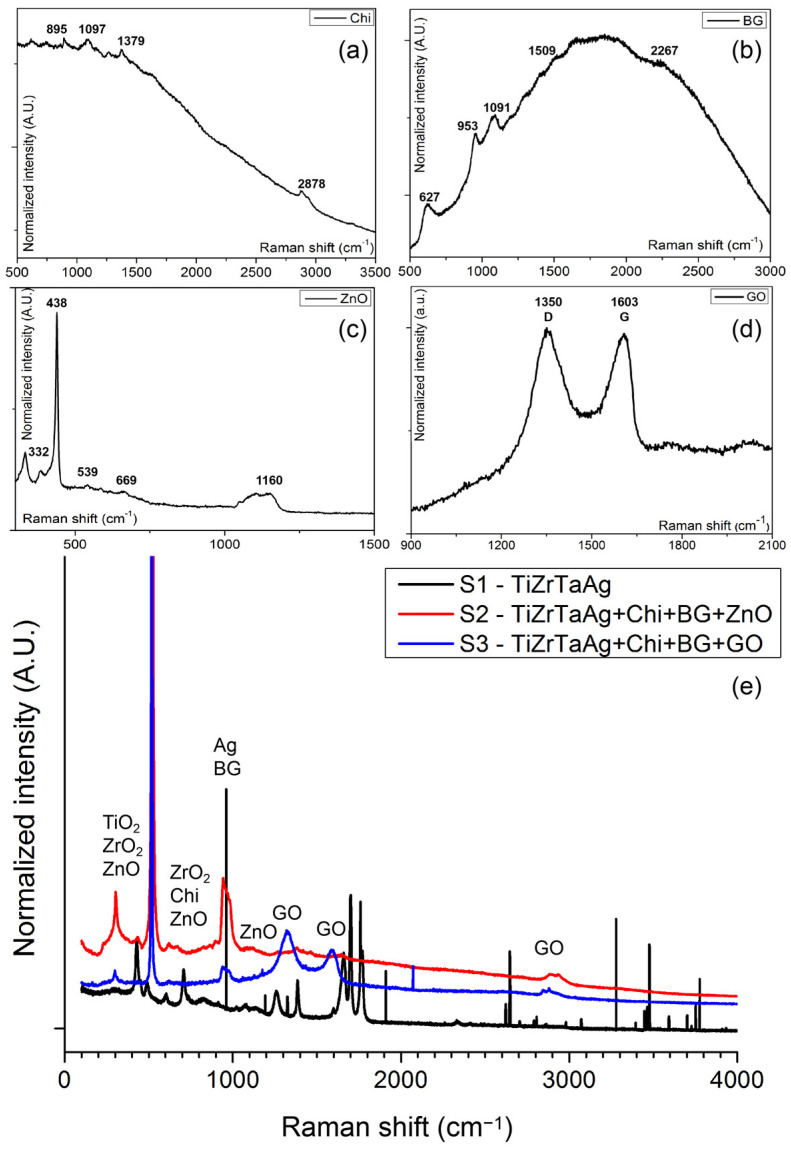
Raman spectra for analyzed samples: (**a**) Chi; (**b**) BG; (**c**) ZnO; (**d**) GO; and (**e**) S1–S3.

The micro-Raman spectrograms recorded on the substrate, on the coating precursors, and on the samples are presented in Figure 3, and prove the presence of all the characteristic peaks of the vibration bands of the components (Table 4). 

For chitosan films, the primary signals observed in Figure 3a were located at 2937 cm^−1^ and 2878 cm^−1^, related to the stretching vibration v(CH2); 1631 cm^−1^ designated for the bending vibration within the δ(NH2) plane; 1380 cm^−1^ linked to combined in-plane bending vibrations of various groups δ(CH2), δ(CH), δ(OH); and at 1098 cm^−1^ related to combined vibrations of groups v(C–O–C) + v(φ) + v(C–HO) + v(C–CH2) + δ(CH) − ρ(CH2) + ρ(CH3). At 896 cm^−1^, a medium intensity absorption was observed, which aligns with the findings in the literature [29]. The addition of 0.25% wt.% of ZnO in Chi films increased absorptions at 1107 cm^−1^ and 670 cm^−1^. In Figure 3e, the position of the Raman peaks observed for sample S2 are in similar positions to those recorded in Figure 3a–c for unpressed chitosan (1277, 1380, 1631 cm^−1^) and BG powders (628, 954, 1092, 1301 cm^−1^), as well as the pressed ZnO powder (220, 323, 438, 670 cm^−1^), highlighting an efficient transfer of the target composition used in the MAPLE process. The Raman spectrogram recorded on sample S3 shows all peaks associated with the precursor materials and were identified as Chi 1277, 1380, and 1631 cm^−1^ and graphene oxide (Figure 3d) 1350, 1603, and 2900 cm^−1^. For S3, the 2D peak position for GO is 2900 cm^−1^, and the ratio ID/IG is 1.18. From the literature data, the classical Raman spectrum of GO is identified using a G band at ca. 1605 cm^−1^, which corresponds to the sp2 C atoms, and a D band at 1353 cm^−1^ [30,31,32,33], and our results are in good concordance with these references (Figure 3d). Peaks associated with the oxides formed on the substrate were identified for TiO_2_ (240, 440, and 310 cm^−1^) and ZrO_2_ (475, 615, and 760 cm^−1^) on the uncoated sample.

### 3.5. Vickers Hardness

It has been discovered that hardness tests are highly helpful for research and development, quality control of manufacturing processes, and material evaluation. Hardness is a way to determine how well a material can resist being indented or scratched. A modification in texture indicates a modification in toughness. The amount of harm is connected to the roughness of the surface, which is a way to quantify the texture/topography of the surfaces employed [34]. Despite being purely empirical, hardness is a measure of ductility and wear resistance and can be connected to tensile strength for many metals. To calculate the microhardness value (HV), five pyramidal indentations were made on each sample using the indenter, measuring the horizontal and vertical diameters. The mean size of the two diagonals left after the indentation of the specimen was performed. The values were transformed to MPa by finding the average of the five indentations [34]. Also, the penetration depths were measured. From Table 5, it can be seen that Vickers hardness was higher for the coated samples (S2, S3) than for the substrate (S1). A film that is less adhered to the substrate may have a hardness value similar to the latter. The higher value obtained for the coated samples, even with the metallic substrate underneath, shows that MAPLE produced dense composite deposits.

**Figure 4:**
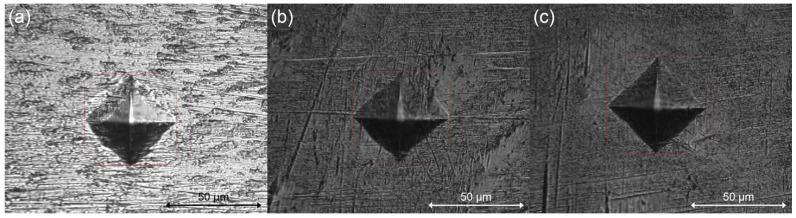
Nanoidentation marks on samples (**a**) S1, (**b**) S2, and (**c**) S3.

In Figure 4 are shown the nanoindentation marks produced on the samples. The average penetration depth of the indenter was 2.3 µm for S1, 5.26 µm S2, and 5.38 µm for S3.

### 3.6. AFM F-Z

**Figure 5:**
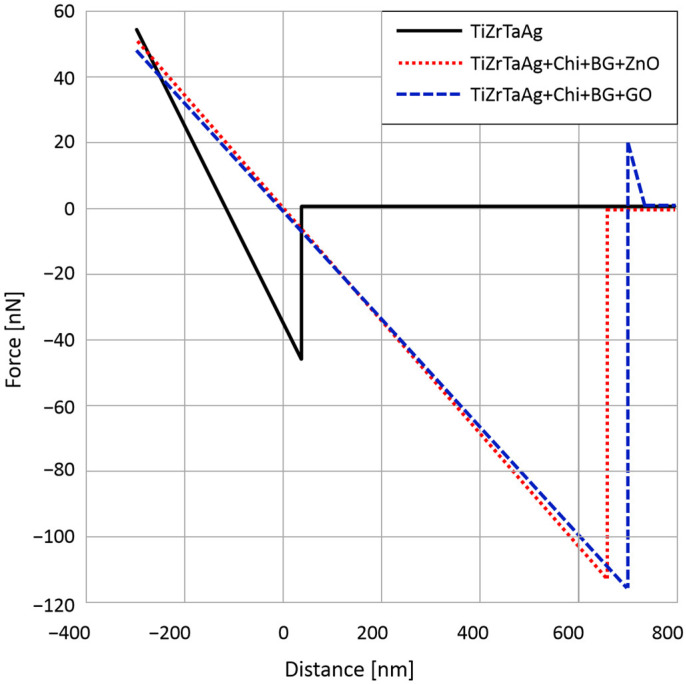
Normalized F-Z curves for the analyzed samples.

The adhesion of the film is an important characteristic that greatly impacts the quality of a coating system. The general shape of the recorded curves (Figure 5) indicates that the deposited films do not break during indentation, having an elastic character. Micro-adhesion forces show differences between samples. For the S1 sample, the adhesion forces are approximately 45 nN and appear over a distance of approximately 70 nm from the sample surface. On sample S1, forces of 110 nN were recorded over a distance of 630 nm. On sample S3, forces of 120 nN were recorded over a distance of approximately 700 nm.

Also, the average roughness of the samples was measured, being 265 nm for S1, 39 nm for S2, and 49 nm for S3. It can be observed that despite the small thickness of the films, the roughness of the coated samples decreases drastically when compared with the uncoated metal surface.

### 3.7. Tafel

**Figure 6:**
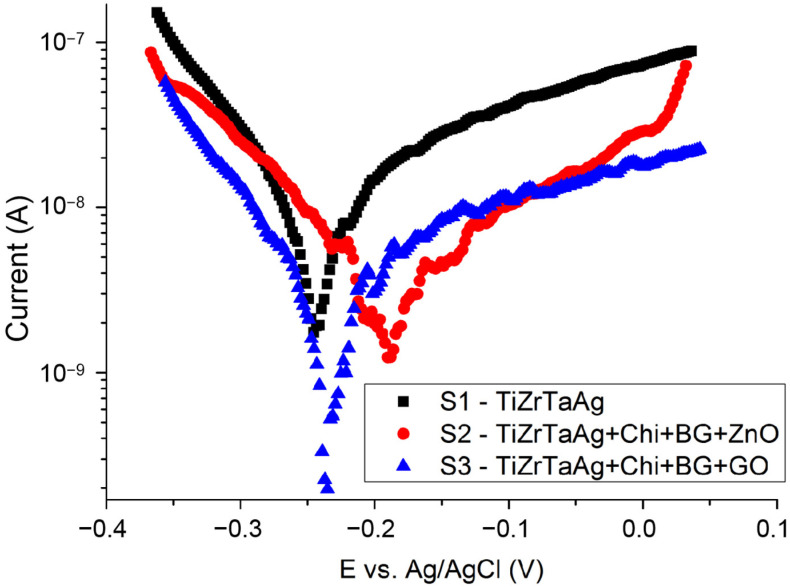
Tafel plots of the analyzed samples.

Potentiodynamic polarization tests were utilized to assess how the samples corroded in a 0.9% NaCl solution. The Tafel plots showing the data are in Figure 6, and the corrosion parameters are listed in Table 6. All samples show remarkable corrosion resistance. The Ecorr values obtained from the coated specimens (S2 and S3) were greater than those from the uncoated samples (S1). Furthermore, for coated samples, the recorded corrosion current density was lower compared to the uncoated samples because of a coating layer that inhibits the direct contact between NaCl and the metallic surface. The corrosion rates of the two types of samples S1 and S2 are close, having the same order of magnitude. Since both coatings contain chitosan, it is likely that their barrier properties are due to a high molecular weight polymer that covers the entire metal surface. Arias et al. [35] pointed out that the presence of Van der Waals forces in chitosan can help in isolating the pores of the coating. 

Samples S2 showed the lowest corrosion rate (six times lower than the speed of the uncoated alloy). The incorporation of GO in chitosan film may act as a barrier against corrosion and prevent the movement of ions in water, as it is impermeable to small molecules [36].

Another important parameter that is linked to corrosion rate is porosity of the samples (Equation (1)), measured after the electrochemical plots, according to a previous paper [37]:

(1) $$P=[(Rp_{substrate}/Rp_{coating})\cdot 10^{-(\left|\Delta E\right|/\beta \alpha )}]\cdot 100\%$$

where *Rp_substrate_* is the polarization resistance of the substrate (uncoated sample), *Rp_coating_* is the polarization resistance of the substrate with a coating (coated samples ***|****ΔE|* is the difference between the substrate and coating corrosion potential, and *βa* is the substrate anodic curve slope. According to Equation (1), the percentage porosities were calculated and are summarized in Table 6.

### 3.8. EIS

**Figure 7:**
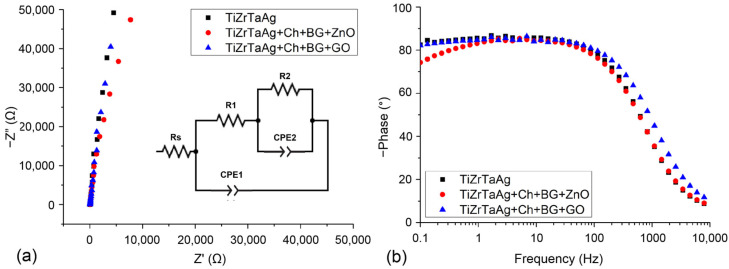
Nyquist (**a**), and Bode Phase (**b**) plots for the analyzed samples. Inset equivalent circuit used to fit the EIS data for the analyzed samples.

The purpose of the EIS measurements was to gather additional information about the electrochemical interfaces by utilizing an equivalent circuit. Each nanocomposite coating displayed capacitive behavior, suggesting that the coatings adhered well and were uniform on the substrate [35]. This conduct may be connected to the insulating property of these coatings. Figure 7a,b are the Nyquist and Bode plot, respectively. In general, a larger radius or semicircle in the Nyquist diagram indicates a greater resistance to charge transfer in the electrochemical process, resulting in a slower corrosion rate and improved corrosion resistance of the sample [38,39]. The Nyquist diagrams in Figure 7a display impedance spectra with a pseudocapacitive shape represented by a tilted straight line. This behavior indicates that either the electron exchanges and reactions are limited, or the oxide layer is highly resistant [40]. Nyquist plots show an increase in spectral amplitude compared to samples without coating, indicating that the presence of the coating enhanced protection for the TiZrTaAg alloy surface. In Figure 7b, it is evident that the capacitive loop of S3 is greater than the capacitive loop of S1. Furthermore, it can be stated that sample S3 displays a high phase angle in Bode diagrams, showing that the Chi + Bg + GO coating increases the corrosion resistance of the sample, which aligns with the findings from the polarization curves.

The EIS data obtained were analyzed by fitting them with the equivalent circuit illustrated in Figure 7 (inset). Table 7 displays the parameter values obtained from the fitting of the equivalent circuit elements using Nova 1.11 software. This equivalent circuit model can generally describe the characteristics of metal samples with coatings [41]. The components selected for the equal circuit are: Rs as the NaCl solution’s resistance, a parallel branch containing the constant phase element (CPE1) linked to the coating capacitance and the coating pore resistance (R1), and another parallel branch containing a constant phase element (CPE2) and a resistance (R2) signifying the electrical characteristics of the metallic substrate, specifically the double layer capacitance and the charge transfer resistance at the metal/electrolyte interface. The CPE is utilized to alter the distribution of relaxation times by taking into account various levels of surface non-uniformity. The capacitance contributes to the overall impedance calculation using the formula:

(2) $$Z_{CPE}=[Q{\left(j\cdot \omega \right)}^{n}{]}^{-1}$$

where Q, j, ω, and n are the pseudo-capacitance or non-ideal capacitance. When n = 1 the system behaves like a pure capacitor [42].

Results of EIS show that R2 is significantly higher than R1 for all samples. Thus, the corrosion performance should be mostly determined with the R2 value. The higher R1 values indicated a lower pore size on the coating, in agreement with SEM and potentiodynamic polarization investigations. As can be seen, the coating increases the value of R2. Furthermore, R2 values of samples with GO in the coating layer are higher than that of the uncoated sample. Therefore, the presence of a thin GO layer would increase the barrier resistance (R2), probably due to an appropriate adhesion provided. The total faradic resistance (Rtot = R2 + R1) of coated samples is higher than that of the uncoated sample. The CPE2 of coated samples are lower than that of the uncoated substrate, confirming an improvement in the barrier effect caused by the coating. The slight decrease in the solution resistance can be attributed to the detachment of the metallic ions form the coating.

The results of impedance spectroscopy confirm the results obtained with the polarization test, and a comparison with the values obtained from EIS and Tafel curves indicates that both techniques yield a similar estimate.

## 4. Conclusions

Two types of coatings deposited using the MAPLE technique on TiZrTaAg substrates were prepared using chitosan, bioglass, ZnO, and GO, and the samples were further characterized using FTIR, SEM-EDX, ellipsometry, micro-Raman, and microhardness. 

The adhesion forces determined using AFM showed no major differences between the coatings. FTIR and micro-Raman revealed the functional groups for each element, with the samples containing hybrid coatings having the same functional groups as each individual component hinting that the MAPLE technique was suitable for composite film deposition. 

From SEM coupled with EDX, the morphology of the coatings was highlighted, showing that the films uniformly covered the substrate and had similar morphologies. In addition to the individual elements existing in the composition of the alloy (Ti, Ta, Zr, Ag), the elements corresponding to the coatings were highlighted using the surface mapping, like O for oxides formed on the substrate and Si, Na, C, O, Mg, and Ca for the coated samples. Using ellipsometry, the thickness of the film was measured, being between 25 and 34 nm. The microhardness of the samples put in evidence that the film deposited on the surface presents higher values in Vickers hardness for the coated samples than for the uncovered one.

Potentiodynamic polarization tests were used to measure the corrosion behavior in 0.9% NaCl. The substrate showed good corrosion resistance, which was further improved after the deposition of the coatings. The film of chitosan inhibited the direct contact between NaCl and the metallic surface. The corrosion rates of the two types of coated samples are in the same order of magnitude. The porosity of the films was also determined. From Nyquist diagrams, we conclude that the presence of the coating on the samples improved the protection of the surface TiZrTaAg alloy. 

All of the characterizations that have been carried out recommend this type of coating from a structural and morphological point of view, considering corrosion behavior to be an important step for future use of these types of coatings as scaffold materials or in implantology. However, the next step worth taking is the testing of these types of coatings from a biological point of view, considering cell viability and proliferation, and considering the fact that porous surfaces favor the growth of osteoblast cells.

## Figures and Tables

**Table 1:** Composition of analyzed samples.

Sample	Composition	Substrate
S1	TiTaZrAg	TiTaZrAg
S2	1% *w*/*v* Chi + 0.25% *w*/*v* BG + 0.25% *w*/*v* ZnO	TiTaZrAg
S3	1% *w*/*v* Chi + 0.25% *w*/*v* BG + 3% *w*/*v* GO	TiTaZrAg

**Table 2:** Elemental composition [% at. wt.] for analyzed samples.

Element vs. Sample	C	O	Na	Si	P	Ca	Ti	Zn	Zr	Ag	Ta
S1	1.20	47.40	0.00	0.00	0.00	0.00	35.83	0.00	11.48	1.68	2.41
S2	8.05	22.85	0.64	0.42	0.18	0.19	57.09	1.47	8.3	0.92	1.27
S3	10.49	22.47	0.19	0.14	0.19	0.12	55.43	0.00	8.91	0.84	1.22

**Table 3:** Film thickness for analyzed samples.

Sample	h (nm)
S2	26.19 ± 1.696
S3	33.42 ± 0.688

**Table 4:** Peak positions for analyzed samples.

Component	Peak (cm^−1^)
Chi	896; 1098; 1277; 1380; 1467; 1631; 2237; 2878; 2937; 3186; 3282; 3395
BG	628; 954; 1092; 1301; 1509; 2268; 2391
ZnO	332; 382; 438; 539; 670; 1107; 1159
TiO_2_	240; 440; 310
ZrO_2_	475; 615; 760
GO	1350; 1603; 2900

**Table 5:** Vickers hardness values and penetration depth for the analyzed samples.

	Vickers Hardness [MPa]			Penetration Depth [µm]		
Applied Force [N]	Sample			Sample		
	S1	S2	S3	S1	S2	S3
0.5	4722.8	5092.4	5208.2	1.4	1.6	1.1
1	4646.3	5036.5	5135.4	3.3	3.1	2.5
2	4752.8	4924.6	4944.4	5.1	4.8	3.6
3	4721.9	4886.4	5122.6	7.2	7	5
5	4712.2	4892.2	5138.3	9.3	10.4	8.2

**Table 6:** Corrosion parameters calculated from the Tafel plots.

Element	S1	S2	S3
Ba [V/dec]	0.174	0.150	0.92
Bc [V/dec]	0.467	0.271	0.239
Ecorr [V]	−0.256	−0.206	−0.240
icorr [nA/cm^2^]	21.55	16.6	3.6
Corrosion rate [μm/year]	0.82	0.19	0.13
Polarization resistance [MΩ]	2.54	6.53	7.99
Porosity %		77.18	32.8

**Table 7:** Parameters of equivalent circuit elements.

Samples	R_s_ [Ω cm^2^]	R1 [kΩ cm^2^]	CPE1 [μF/cm^2^]	n	R2 [kΩ cm^2^]	CPE 2 [μF/cm^2^]	n
S1	38.8	20.7	6.58	0.98	3.32 × 10^3^	6.86	0.91
S2	36.6	461	8.15	0.86	7.12 × 10^3^	3.39	0.86
S3	37.6	718	1.86	0.91	9.14 × 10^3^	2.72	0.88

## Data Availability

The data used to support the findings of this study are available from the corresponding author upon request.
